# Chemotherapy combined with bevacizumab for the treatment of advanced lung adenocarcinoma cancer harboring EGFR‐ANXA2, EGFR‐RAD51, ATR and BRCA2 mutations: A case report

**DOI:** 10.1111/1759-7714.13286

**Published:** 2019-12-22

**Authors:** Rui Zhong, Hui Li, Yanling Liu, Shuang Zhang, Jingjing Liu, Zhicheng Huang, Ying Cheng

**Affiliations:** ^1^ Translational Cancer Research Lab Jilin Cancer Hospital Changchun China; ^2^ Department of Medical Breast Oncology Jilin Cancer Hospital Changchun China; ^3^ Department of Medical Thoracic Oncology Jilin Cancer Hospital Changchun China; ^4^ Department of Radiation Jilin Cancer Hospital Changchun China

**Keywords:** Bevacizumab, chemotherapy, EGFR fusion, NGS, NSCLC

## Abstract

Here, we report a case of a 36‐year‐old female patient with metastatic non‐small cell lung cancer (NSCLC) harboring EGFR‐ANXA2 and EGFR‐RAD51 double fusion mutations with BRCA2 (nonsense mutation of exon 11) and ATR mutations (Exon 44 variable shear mutation) identified by next generation sequencing (NGS). The efficacy was significantly improved after lobaplatin combined with pemetrexed, temozolomide and bevacizumab. This is the first report of a novel mutation type EGFR‐ANXA2, as well as double EGFR fusion mutations in advanced lung adenocarcinoma. Furthermore, platinum‐based chemotherapy plus bevacizumab rather than targeted therapy showed favorable effects in this patient, providing a novel therapeutic conception for patients, even with multidriver mutations.

## Introduction

Next generation sequencing (NGS) is a powerful tool which identifies complex mutations, including gene deletion, point mutations, small indels as well as genetic fusion. Nowadays, identification of EGFR additional mechanisms through NGS on tumor samples (such as tumor tissue and plasma) document the importance of EGFR signaling in the pathogenesis of lung cancer.^1^, ^2^ EGFR fusion mutations are rare events,^3^, ^4^ and four types have been previously reported in lung cancer, including EGFR‐RAD51 and EGFR‐PURB, EGFR‐TNS3 and EGFR‐ZCCHC6.^5^, ^6^ Some reports showed that patients with EGFR‐RAD51 or EGFR‐PURB experienced a remarkable tumor response to EGFR TKI such as afatinib,^7^ elotinib, or chemotherapy such as carboplatin and pemetrexed.^6^ However, there is no report in the literature on EGFR‐ANXA2 and EGFR‐RAD51 double fusion mutations in terms of discovery and treatment. Here, we present a single case of an NSCLC patient harboring EGFR‐ANXA2 and EGFR‐ RAD51 double fusion mutations with ATR and BRCA2 mutations which is the first to be reported to the best of our knowledge. We determined that the patient received significant clinical benefit from the treatment of lobaplatin combined with pemetrexed, temozolomide and bevacizumab.

## Case report

**Figure 1:**
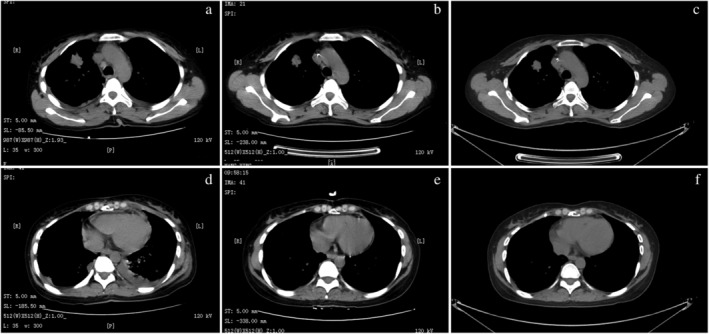
Computed tomography (CT) of the chest before and after four‐months treatment using chemotherapy combined with bevacizumab. Lung tumor regression is shown in (**a**) and (**b**); the pericardial effusion alteration is shown in (**c**) and (**d**).

**Figure 2:**
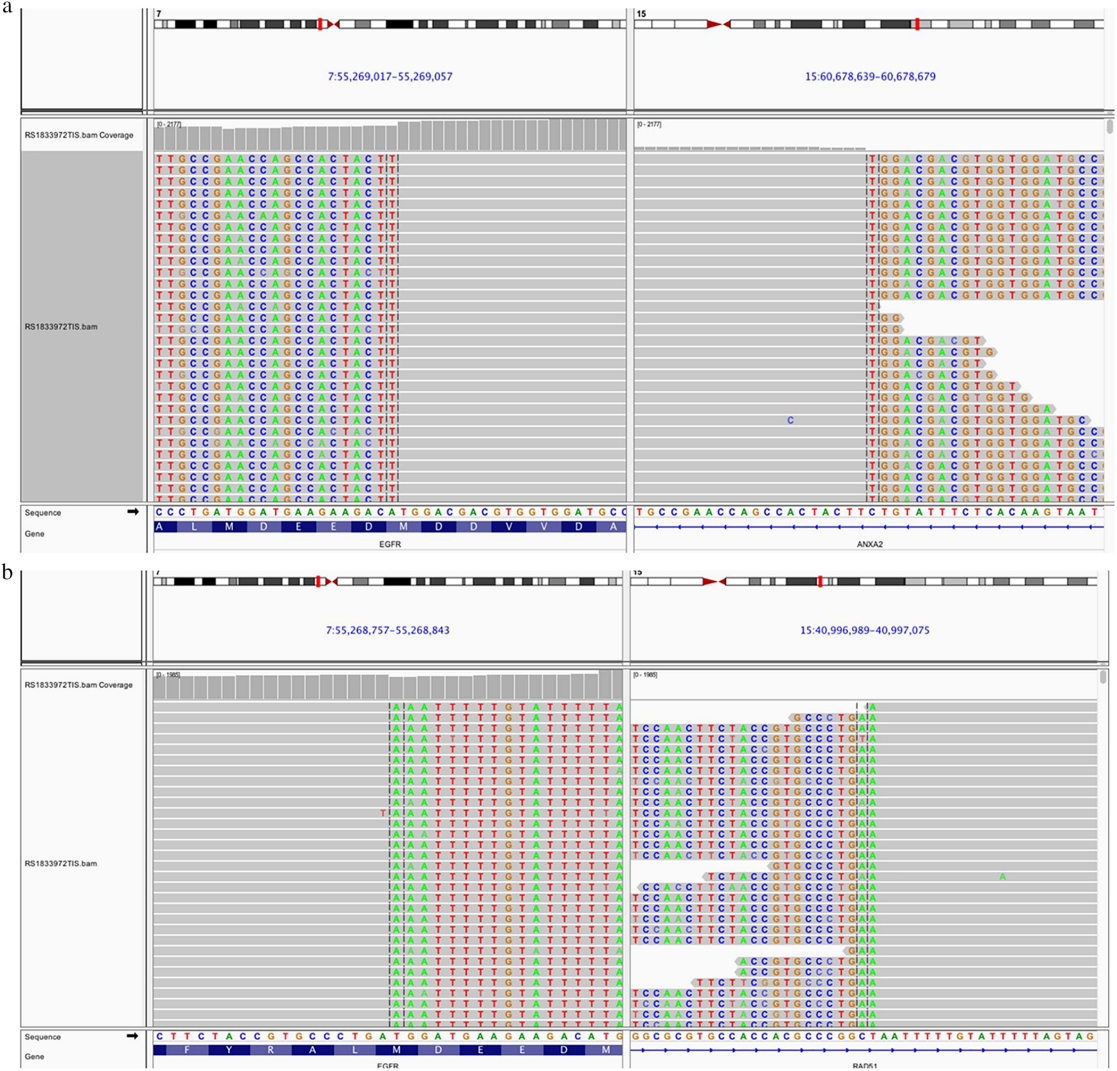
The Integrative Genomics Viewer snapshot of EGFR‐ANXA2 (**a**) and EGFR‐RAD51 (**b**).

A 36‐year‐old never smoking woman complained of symptoms of cough and difficulty breathing. She was admitted to our hospital in July 2018. Thoracic CT scan showed a 2.6 cm right lung mass and bilateral pleural effusion (Fig 1a,d) and the patient was diagnosed with lung adenocarcinoma (cT1bNxM1 stage IV) with brain metastasis, double lung metastasis, sternal metastasis, pericardial metastasis, left pleural metastasis, bilateral pleural effusion and pericardial effusion in September 2018. The patient received fluorouracil (250 mg), lobaplatin (10 mg), and bevacizumab (100 mg) on three occasions for thoracic perfusion. Following treatment, the bilateral pleural effusion was significantly reduced. In order to seek more effective treatment, genetic testing was performed in October 2018 using tissue biopsy derived from a pulmonary nodule through a panel‐targeted NGS (520 cancer‐related genes). The results of the test showed a novel EGFR arrangement between EGFR and ANXA2 (the mutation abundance was 27.49%, Fig 2a), a EGFR rearrangement between EGFR and RAD51 (the mutation abundance was 23.88%, Fig 2b) as well as ATR exon 44 variable shear mutation (the mutation abundance was 3.14%) and BRCA2 exon 19 nonsense mutation (the mutation abundance was 3.75%). The TMB of the case was 10.3 mutations/megabase. Based upon these results, the patient received the combination of pemetrexed (755 mg), lobaplatin (45.3 mg), bevacizumab (375 mg) and temozolomide (1000 mg) for six cycles followed by pemetrexed (755 mg), bevacizumab (375 mg) and temozolomide (1000 mg) for maintenance treatment. Side effects of treatment were third degree of myelosuppression, and I degree of gastrointestinal reaction.

A first CT evaluation performed two cycles post‐treatment showed the density of the major consolidation had decreased remarkably (2.1 cm, Fig 1b). Furthermore, the bilateral pleural (Fig 1e) and pericardial effusions had disappeared and brain metastasis had decreased. The last follow‐up imaging assessment performed six cycles after treatment initiation showed a continuous decrease in density of the major consolidation (2.0 cm, Fig 1c). In addition, pleural effusion (Fig 1f) and pericardial effusion were not present, brain metastasis had disappeared and no new lesions were visible. According to the Response Evaluation Criteria in Solid Tumors (RECIST) guidelines (version 1.1), local lesions were stable disease (SD), and overall efficacy were stable disease (SD).

## Discussion

NGS is a high‐throughput sequencing method that simultaneously sequences multiple target genomic regions in multiple samples with high‐precision and high‐sensitivity. Here, we used a 520 cancer‐related gene panel with sensitivity of 1%–2% and the sequencing depth was 1000x. Since EGFR‐ANXA2 concomitant with EGFR‐RAD51 were novel and the abundance of ATR and BRCA2 was relatively low in this case, we suspected that only high‐throughput plus high sensitivity mutation analysis would provide the potential to determine such gene mutations.

Previous studies showed that patients with EGFR‐fusion mutations experienced a remarkable tumor response to carboplatin/pemetrexed or EGFR‐TKI therapies.^6^, ^7^ However, few investigations have focused on the EGFR double fusion mutations concomitant with other driver mutations, leaving the optional therapy unclear. In our case, the breakpoint positions of EGFR‐RAD51 were near EGFR7q21.11 and RAD5115q21.2, which included EGFR exon 1–25 (1001 amino acids) and RAD51 exon 5–10 (224 amino acids). The breakpoint positions of EGFR‐ANXA2 were near EGFR 7q21.11 and ANXA2 15q21.2, which included EGFR exon 26–28 (203 amino acids) and ANXA2 exon 2–13 (342 amino acids). The transcription direction of EGFR‐RAD51 started with 5’ EGFR and was followed by 3’ RAD51. However, for EGFR‐ANXA2, both EGFR and ANXA2 started from 3' end. Although the EGFR‐ANXA2 could form precursor RNA, this 3′ end followed by 3′ end was hardly translated into functional protein. In addition, individual gene of EGFR ANXA2 did not contain a 5′UTR region, proving further evidence that the fusion mutation may not be functional.

It has been reported that patients with BRCA1/2 mutation are sensitive to platinum‐based chemotherapy^8^, ^9^ or bevacizumab,^10^, ^11^ and ATR loss‐of‐function mutations are sensitive to platinum or anti‐PD‐1/PD‐L1 immunotherapy.^12^, ^13^ In addition, BRCA2 and ATR are involved in homologous recombination deficiency (HRD)^14^, ^15^ and patients with HRD are sensitive to platinum‐based chemotherapy or PARP inhibitor.^16^, ^17^ We assumed our case might belong to the HRD group of patients. Previous studies indicated that BRCA1/2 and ATR mutations are usually associated with high TMB.^18^, ^19^ In our case, the TMB was relatively high (10.3 mutations/megabase with cutoff as 10) and PD‐L1 expression status was unknown. Considering the complex presence of multi‐driver mutations and various regimen strategies, we selected chemotherapy plus bevacizumab as a cost‐effective and feasible regimen for the patient rather than EGFR‐TKI, PARP inhibitor or immune‐targeted drugs. Furthermore, we used temozolomide because the patient had brain metastases. Our data showed that a conventional drug regimen such as chemotherapy or anti‐angiogenesis was a good choice for complex and concomitant mutations, especially for multi‐driver mutations with novel mutations.

With the application of NGS in clinical practice, more and more rare mutations and concomitant driver‐mutations beyond the well known single driver mutation (EGFR, ALK, ROS1) will be discovered. How to treat those patients with individualized targeted therapy combination to reach best efficacy is challenging. To our knowledge, this is the first report on novel mutation types, EGFR double fusion mutations plus BRCA and ATR mutations in a Chinese patient with lung adenocarcinoma. Second, we found chemotherapy plus bevacizumab had a good effect in this patient, providing a novel therapeutic concept to treat such a patient. Since it is hard to carry out clinical trials due to patient size in order to determine an effective treatment regimen for such a rare mutation, our case report provides valuable real‐word evidence. In future, fundamental research on such rare mutations in terms of mechanism of tumorigenesis and disease progression will be urgently needed.

## Disclosure

The authors report no conflicts of interest in this work.
